# Comparison of Laboratory and Radiological Findings of Pregnant and Non-Pregnant Women with Covid-19

**DOI:** 10.1055/s-0041-1726054

**Published:** 2021-04-15

**Authors:** Kadir Burak Ozer, Onder Sakin, Kazibe Koyuncu, Berk Cimenoglu, Recep Demirhan

**Affiliations:** 1 Department of Thoracic Surgery, Kartal Training and Research Hospital, University of Health Sciences, Istanbul, Turkey; 2 Department of Obstetrics and Gynecology, Kartal Training and Research Hospital, University of Health Sciences, Istanbul, Turkey

**Keywords:** covid-19, pregnancy, laboratory, imaging studies

## Abstract

**Objective** Covid-19 became a pandemic, and researchers have not been able to establish a treatment algorithm. The pregnant population is also another concern for health care professionals. There are physiological changes related to pregnancy that result in different laboratory levels, radiological findings and disease progression. The goal of the present article is to determine whether the laboratory results and radiological findings were different in non-pregnant women (NPWs) of reproductive age and pregnant women (PWs) diagnosed with the Covid-19 infection.

**Methods** Out of 34 patients, 15 (44.11%) PWs and 19 (55.8%) NPWs were included in the study. Age, comorbidities, complaints, vitals, respiratory rates, computed tomography (CT) findings and stages, as well as laboratory parameters, were recorded from the hospital database.

**Results** The mean age of the PWs was of 27.6 ±  0.99 years, and that of the NPWs was of 37.63 ±  2.00; when age was compared between the groups, a statistically significant difference ( *p* = 0.001) was found. The mean systolic blood pressure of the PWs was of 116.53 ±  11.35, and that of the NPWs was of 125.53 ±  13.00, and their difference was statistically significant ( *p* = 0.05). The difference in the minimum respiratory rates of the patients was also statistically significant ( *p* = 0.05). The platelet levels observed among the PWs with Covid-19 were lower than those of the NPWs (185.40 ±  39.09 × 109/mcL and 232.00 ±  71.04 × 109/mcL respectively; *p* = 0.05). The mean D-dimer value of the PWs was lower in comparison to that of the NPWs ( *p* < 0.05).

**Conclusion** The laboratory findings and imaging studies may differ between pregnant and non-pregnant populations. It is important to properly interpret these studies. Future studies with a higher number of patients are required to confirm these preliminary data.

## Introduction

The Covid-19 pandemic was declared by the World Health Organization (WHO) on March 11, 2020. As of today, approximately 4.115 million Covid-19 cases have been reported worldwide, and 280 thousand of them were fatal. 1 The virus causing symptoms ranging from flu-like symptoms to signs of severe respiratory failure similar to severe acute respiratory syndrome (SARS) and Middle-East respiratory syndrome (MERS) has been announced to be a novel coronavirus (2019-nCoV) that had never been previously detected in humans. Studies 2 have demonstrated that this type of coronavirus has 96.3% of homology with bat SARS-like coronavirus (BatCoV RaTG13), and bats can be natural hosts for 2019-nCoV.

The symptoms reported to be associated with Covid-19 infection are cough (50%), fever (43%), myalgia (36%), headache (34%), dyspnea (29%), sore throat (20%), diarrhea (19%), nausea/vomiting (12%), loss of smell/taste (10%), abdominal pain (10%), and rhinorrhea (10%). 3 Moreover, particular laboratory results were found to be related to poor prognosis, and they are listed as lymphopenia, thrombocytopenia, elevated liver enzymes, elevated lactate dehydrogenase (LDH), elevated inflammatory markers (such as C-reactive protein [CRP] and ferritin), elevated D-dimer (> 1 mcg/mL), elevated prothrombin time (PT), elevated troponin, elevated creatine phosphokinase (CPK), and acute kidney injury. 4 5 6 These changes were not subclassified into specific populations.

The laboratory results of pregnant women (PWs) differ from those of non-pregnant women (NPWs); they even differ regarding pregnant women in different trimesters. For example, the leukocyte count and the levels of alkaline phosphatase continuously rise during a normal pregnancy. Similarly, D-dimer values approximately double during mid-pregnancy. Several hormones and coagulation factors are also known to increase substantially. Furthermore, inflammation markers such as ferritin and CRP levels were shown to rise. Unless these normal, pregnancy-related alterations are taken into account when evaluating the laboratory results of PWs, many of the physiological adaptations of pregnancy can be misinterpreted as pathological, or they may mask the diagnosis of a disease process.

Pregnancy causes specific physiological changes, and laboratory results differ during pregnancy. Therefore, it is important to know these changes to properly interpret these studies. Dyspnea and discomfort in breathing are common during pregnancy, and are also common in cases of Covid-19 infection. Moreover, the diaphragm is elevated since the first trimester, and it rises up to 4 cm, and the diameter of the chest can increase by 2 cm or more. 7 These changes could make it hard to clarify the radiological findings in PWs infected with Covid-19. Respiratory rates were shown to be unchanged. 8 Systolic blood pressure tends to decrease between the 12th and 19th weeks of gestation, then tend to progressively rise until the 40th week. Similarly, diastolic blood pressure decreases and increases throughout pregnancy. Notwithstanding the knowledge of these normal, pregnancy-related alterations, laboratory results and radiological findings could lead to misdiagnosis and alter the management of the pregnancy. The objective of the present study is to compare laboratory parameters and radiological findings between PWs and NPWs of reproductive age hospitalized for Covid-19 infection.

## Methods

The present retrospective cross-sectional study was conducted between March 18 and May 1st, 2020, in a Turkish tertiary healthcare center which is one of the country's first pandemic hospitals. A total of 34 patients treated in the pandemic wards were included in the study, 15 of whom were pregnant. Age, comorbid diseases, complaints, systolic and diastolic arterial blood pressures and the minimum and maximum values of oxygen saturation, respiratory rates, computed tomography (CT) findings and stages, and laboratory parameters, such as CRP, D-dimer, neutrophil, lymphocyte, leukocyte and platelet counts, were collected from hospital records. The present study included PWs and NPWs of reproductive age with positive Covid-19 test results who were submitted to inpatient treatment.

At the first visit to the emergency room or Covid-19 outpatient clinics, PWs and NPWs with fever, low oxygen saturation (< 93% mmHg in the air in the room), presence of comorbidities, and asymptomatic PWs in the last trimester were hospitalized in pandemic wards. Female patients under the age of 18, those not of childbearing age, all male patients, and patients without CT findings specific to Covid-19 disease or who were not positive after a polymerase chain reaction (PCR) test were excluded from the study. All retrospective data were examined, and informed consent was obtained from the patients and their relatives. The present study was approved by the institutional Ethics Committee and the Ministry of Health, General Directorate of Health Services (2020/514/176/17).

**Fig. 1:**
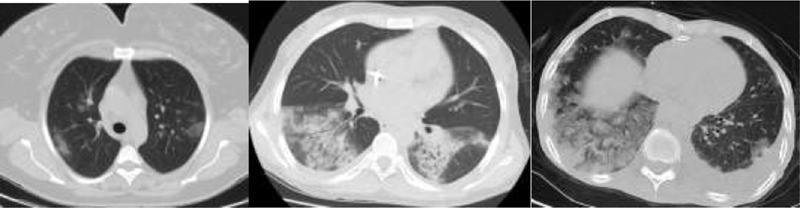
Stages of Covid-19 infection according to computed tomography findings ( **1A** : early stage; **1B** : progressive stage; **1C** : severe stage – diffuse consolidation).

The PWs underwent CT scans after delivery, and the NPWS, at the time of hospitalization. The lesions detected on the CT scans were divided into three stages: early stage, progressive stage, and severe stage, according to their locations, density, presence of air bronchograms ,and multiple-lobe involvement. 9 The early stage is characterized by the ground-glass opacity (GGO), which is common in Covid-19 disease, located in the peripheral and subpleural area. 9 10 The GGO is described as an irregular-shaped shadow image that reduces the density of the lung tissue. 10 In the progressive stage, areas of inflammation that are more intense and apparent than the GGO, as well as centrally-located consolidations that contain air bronchograms, are observed. The CT finding in which bilateral and diffuse intensely-consolidated areas in both lungs acquire a marble-like appearance is considered as the severe stage. 9 In the present study, we classified the CT findings of all patients according to their stages, and evaluated them through a statistical analysis ( Fig. 1 ).

All retrospectively-collected data were analyzed with the Statistical Package for the Social Sciences (SPSS, IBM Corp., Armonk, NY, US) software, version 22.0. the relationships among the values found for the PWs and NPWs were evaluated using the Chi-Squared test, and the analysis of the continuous variables was performed through the Student *t* -test. One-way analysis of variance (ANOVA) was performed for the evaluation of the relationships among more than two groups. The correlations of two continuous variables were determined by the Pearson correlation test. Values of *p* lower than 0.05 were considered statistically significant.

## Results

A total of 34 patients, 15 (44.11%) PWs and 19 (55.8%) NPWs, were included in the present study. The mean age of the patients was of 33.7 ±  1.49). The mean age of PWs was of 27.6 ±  0.999, while the mean age of the NPWs was of 37.63 ±  2.009. The difference between the average ages of these two groups was statistically significant ( *p* = 0.001). The mean gestational week among the PWs was 31 ±  7.92 (11 to 40 weeks). One of the PWs had allergic asthma and another had hypothyroidism. Among the NPWs, 4 had hypertension, 3 had diabetes mellitus, 2 had allergic asthma, and 1 had chronic renal failure that did not require dialysis. The most common abnormal vital sign in the whole sample was fever. The analysis of the vital signs of the patients is outlined in table 1 . The maximum and minimum values of all vital parameters were recorded, and a statistical comparison was performed between the PWs and NPWs. According to this analysis, a significant difference was found between the maximum values of arterial blood pressure ( *p* = 0.039) and the minimum respiratory rates per minute ( *p* = 0.013). There was no significant difference regarding the other vital parameters of two groups ( Table 1 ).

**Table 1:** Statistical analysis of the vital parameters of the two study groups

	Pregnant		Non-pregnant		*p-value*
	Mean ± standard deviation	(Minimum–maximjum)	Mean ± standard deviation	(Minimum–maximum)	
Systolic blood pressure	116.53 ± 11.351 mm/Hg	148–85 mm/Hg	125.53 ± 13.006 mm/Hg	150–90 mm/Hg	**< 0.05**
Diastolic blood pressure	76.00 ± 11.212 mm/Hg	110–60 mm/Hg	79.47 ± 10.260 mm/Hg	100–50 mm/Hg	> 0.05
Oxygen saturation (maximum)	98.53 ± 0.915%	99–94%	98.21 ± 0.631%	99–93%	> 0.05
Oxygen saturation (minimum)	96.20 ± 6.213%	96–89%	96.58 ± 1.677%	94–88%	> 0.05
Fever (maximum)	37.247 ± 0.7039°	38.–36.2°	37.405 ± 0.8508°	39.–36.5°	> 0.05
Fever (minimum)	36.487 ± 0.6490°	37.5– 35.5°	36.558 ± 0.4682°	37.3– 35.7°	> 0.05
Respiratory rate (maximum)	21.531. 959 per minute	27–20	20.05 2.592 per minute	27–17	> 0.05
Respiratory rate (minimum)	19.73 1.981 per minute	26–18	19.73 1.981 per minute	20–16	**< 0.05**

The mean leukocyte count of the PWs was of 6,486.6 ±  2,86128 × 103/mcl; the mean lymphocyte count was of 896 ± 235.184 mm ^3^ ; the neutrophil count was of 4,293.3 ±  1,338.158 mm ^3^ ; and the mean platelet count was of 185.4 ±  39.089 x109/mcL. When the average laboratory results of the NPWs were evaluated, the leukocyte count was of 1,4473.6 ±  32,941.292 × 103/mcl, the lymphocyte count was of 1,488.4 ±  2,596.513) mm ^3^ , the neutrophil count was of 12,989.4 ±  30,327.397 mm ^3^ , and the platelet count was of 232 ±  71,040 x109/mcL. Although the platelet counts were within normal limits in both groups, the fact that the PWs had lower platelet counts was found to be statistically significant ( *p* = 0.026) ( Table 2 ).

**Table 2:** Statistical analysis of laboratory parameters and radiological findings

	Pregnant	Non-pregnant	*p* -value
Leukocytes (x103/mcL)	6,486.67 ± 2,861.285	14,473.68 ± 32,941.292	> 0.05
Lymphocytes (mm ^3^ )	896.00 ± 235.184	1488.42 ± 2596.513	> 0.05
Neutrophils (mm ^3^ )	4293.33 ± 1338.158	12989.47 ± 30327.397	> 0.05
Platelets (x109/mcL)	185.40 ± 39.089	232.00 ± 71.040	< 0.05
C-reactive protein (mg/dL)	46.107 ± 53.3711	44.842 ± 71.2431	> 0.05
D-dimer (ng/mL)	962.00 ± 523.644	3732.11 ± 6605.985	< 0.05
Computed tomography findings – early stage: n (%)	2 (13.3%)	10 (52.63%)	> 0.05
Computed tomography findings – progressive stage: n (%)	4 (26.6%)	5 (26.3%)	> 0.05
Computed tomography findings – severe stage: n (%)	2 (13.3%)	1 (5.2%)	> 0.05

**Fig. 2:**
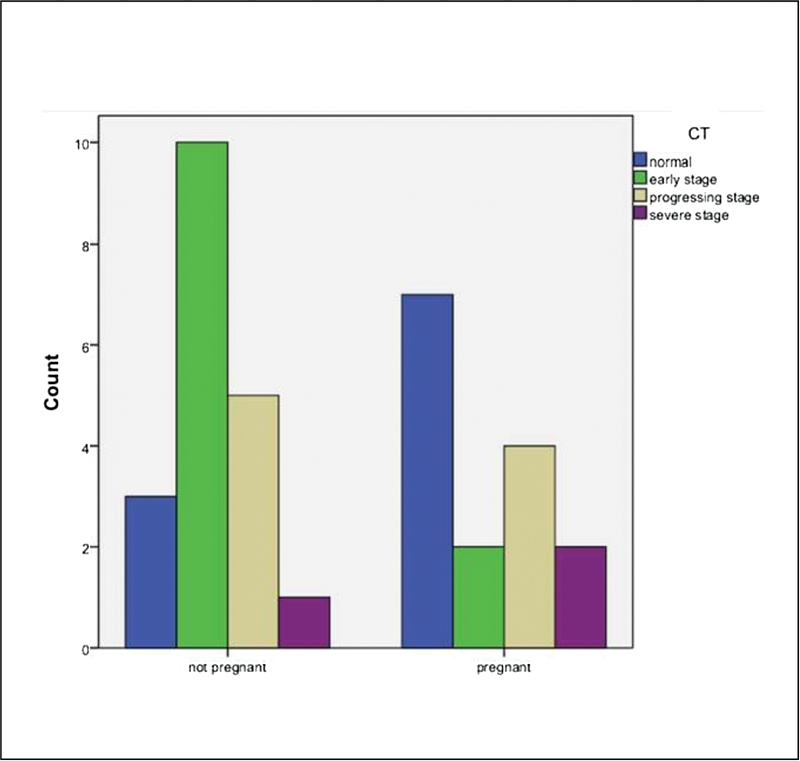
Distribution of stages according to computed tomography findings.

The mean CRP values of the PWs at the time of admission was of 46.1053.371 mg/dl (minimum: 3 mg/dl; maximum: 148 mg/dl), and, regarding the NPWs, it was of 44.8 ±  71.243) mg/dl (minimum: 235 mg/dl; maximum: 2,1 mg/dl). The difference between these two groups was not statistically significant ( *p* > 0.05). The mean value of the D-dimer of the PWs with Covid-19 was of 962 ±  523.644 ng/ml (minimum: 540 ng/ml; maximum: 2,560 ng/ml), and, regarding the NPWs, it was of 3,732.1 ±  6,605.985 ng/ml (minimum: 200 ng/ml; maximum: 30,000 ng/ml). This difference was found to be statistically significant ( *p* < 0.05). It was determined that the CRP and D-dimer values of all patients were positively correlated with each other. According to the postnatal CT findings of PWs with Covid-19, 2 (13.3%) patients were in the early stage, 4 (26.6%) were in the progressive stage, and 2 (13.3%) were in the severe stage ( Fig. 1 ). No abnormal CT findings were detected in 7 (46.6%) patients. We observed that 10 (52.63%) NPWs with Covid-19 had early-stage CT findings, 5 (26.3%) had progressive-stage findings, and 1 (5.2%) had severe-stage findings. In the comparative analysis between the two groups, no significant difference was found in terms of tomography findings ( *p* > 0.05). The CT stages and laboratory and vital parameters were compared, and the relationship between CT stages and D-dimer values among PWs with Covid-19 was statistically significant ( *p* = 0.045) ( Tables 3 and 4 ) ( Fig. 2 ).

**Table 3:** Number of pregnant patients and D-dimer values according to the stages of Covid-19 infection assessed through computed tomography

	Normal	Early stage	Progressive stage	Severe stage
Patients – n (%)	7 (46.6%)	2 (13.3%)	4(26.6%)	2 (13.3%)
D-dimer values (ng/ml) (mean ± standard deviation)	637.14 ± 141.03	925.00 ± 176.77	1,505.00 ± 754.29	1,050.00 ± 212.13

**Table 4:** Number of non-pregnant patients and D-dimer values according to the stages of Covid-19 infection assessed through computed tomography

	Normal	Early stage	Progressive stage	Severe stage
Patients – n (%)	2 (10.5%)	10 (52.6%)	5(26.3%)	1 (5.2%)
D-dimer values (ng/ml) (mean ± standard deviation)	613.33 ± 270.24	1,633.00 ± 1,178.74	9,574.00 ± 11,483.00	4,900.00

A statistically significant difference was found between the CRP values of PWs with CT findings of all 3 stages ( *p* = 0.002). There was no statistically significant difference between the oxygen saturation values of patients in different stages according ot the CT findings ( *p* > 0.05).

## Discussion

The highly-transmissible Covid-19 disease affected the whole world within a few weeks. The morbidity and mortality rates quickly became fairly high. Although the overall mortality rate was lower than that of other coronavirus outbreaks, the sheer number of deaths recorded due to the great number of patients. In the general population, 81% of the cases reported so far are mild, 14% are moderate, and 5% are severe in terms of clinical condition. 11 Huang et al. 2 emphasized that the severity of the disease has a positive correlation with age. In the Covid-19 disease, the mortality rates have been found to be higher, particularly in patients older than 50 years of age. 12 In a review of the studies in which PWs with Covid-19 were followed up, low morbidity and mortality rates were detected in PWs in comparison with the general population. 13 14 15 The fact that pregnant Covid-19 patients were usually younger than 40 years, as is the case in the present study, can be the explanation behind the low morbidity and mortality rates.

We observed that the maximum systolic blood pressure was higher among NPWs. This is related to the presence of history of chronic hypertension in 4/19 of the population of NPWs. In a study conducted by Li et al., 16 the Covid-19 disease was shown to be significantly more aggressive in individuals with preexisting comorbidities. According to their data, the fact that fewer comorbid diseases were found among the PWs with Covid-19 is the probable explanation for the milder course of Covid-19 during pregnancy. Through the comparative analysis of the laboratory parameters of PWs and NPWS with Covid-19, the platelet counts were found to be significantly higher among NPWs, even though they were within the normal range for both groups.

Ranucci et al. 17 determined that the formation of microthrombi in the pulmonary vascular bed was an important factor in the development of acute respiratory distress syndrome (ARDS) among Covid-19 patients. In addition, they did not observe any major thromboembolic events after initiating the anticoagulant treatment in a case series with 16 patients. 17 It could be said that low molecular weight heparin (LMWH) should be used in the prophylaxis, especially when the increased risk of thrombosis risk in cases of Covid-19 is added to the increased risk of hypercoagulopathy during pregnancy. It is known that D-dimer values rise during pregnancy. The normal D-Dimer values reported according to the trimester were of 200 ng/mL to 900 ng/mL in the first trimester, 200 ng/mL to 1600 ng/mL in the second trimester, and 400 ng/mL to 500 ng/ mL in the third trimester. The mean D-dimer level found in the present study was a little higher than expected. Our recommendation is to use LMWH at the prophylaxis dose in infected PWs, and to use the treatment dose in the PWs with high D-dimer levels. Tang et al. 18 stated that patients with significantly high D-dimer levels would benefit from the anticoagulant treatment given at the treatment dose. As a result of the autopsies performed in 5 patients who died from Covid-19, Magro et al. 19 declared that microtrombi tend to join in different vascular zones. In our study, we found that D-dimer values were significantly higher among the NPWs. The lower D-dimer values among PWs may explain their lower mortality and morbidity rates. However, studies with larger samples are required for more precise results.

There was no statistically significant difference in CRP values between PWs and NPWs. The CRP values of the patients who required additional antibiotherapy for conditions such as fever are significantly higher than those of the patients who did not require antibiotherapy. Therefore, a high CRP value is an important factor to take into consideration before initiating antibiotherapy. On the other hand, it should be kept in mind that antibiotherapy should be added to the current treatment protocol, as an increase in CRP values is detected during the follow-up. In the present study, a positive correlation was found between elevated CRP and D-dimer values. Sun et al. 20 also determined a positive correlation between the increase in CRP and D-dimer values.

According to the radiological studies on Covid-19 disease, many disease-specific findings were detected in CT of the thorax, such as the GGO and consolidation. In a study conducted by Wang et al., 9 the patients were staged radiologically according to the CT findings. In the present study, all patients were divided into three different stages according to the CT findings. There was no statistically significant difference regarding the tomographic stages of the PWs and NPWs with Covid-19. However, the differences in CRP and D-dimer values among CT stages were statistically significant ( *p* < 0.05). The highest mean CRP values (130 ±  25.4) were observed in patients in the severe stage , and the highest mean of D-dimer values (1505 ±  754) were observed in patients in the progressive stage. Adding antibiotherapy and anticoagulant treatment according to the CT stage should be considered while planning the treatment protocols for pregnant patients. This will also be clarified with more data.

The limitations of the present study include the small number of the patients, which was due to the low number of PWs diagnosed with Covid-19. This may be related to the better adaptation of PWs to social isolation and sanitary habits. We also wish we had preliminary data to better understand the laboratory results in order to take immediate action for the pandemic process.

## Conclusion

There is very little data on the Covid-19 disease, and information on pregnancy and its consequences is particularly limited. We observed that pregnancy did not have any effect on laboratory values or radiological findings in Covid-19 infection period. Especially in pregnant patients, we recognized that different laboratory results appeared according to tomographic stages. As a result, taking tomographic staging into consideration while creating treatment algorithms for pregnant patients was found to be necessary. On its own, pregnancy was not found to be a poor prognostic factor for Covid-19 disease if the radiological and laboratory findings were evaluated. However, with extensive studies in the future, clearer comments will be made on this subject.
